# Gut microbiome disruption in free-roaming cats: Do antibiotics reduce or restructure zoonotic risk?

**DOI:** 10.1016/j.onehlt.2025.101186

**Published:** 2025-08-29

**Authors:** An Xie, Yiyue Zhang, Zhonghui Tang, Jake M. Robinson, Qiansheng Huang, Jian-qiang Su, Xin Sun

**Affiliations:** a State Key Laboratory of Regional and Urban Ecology, Ningbo Observation and Research Station, Fujian Key Laboratory of Watershed Ecology, Institute of Urban Environment, Chinese Academy of Sciences, Xiamen, China; b University of Chinese Academy of Sciences, Beijing 100049, China; c Zhejiang Key Laboratory of Urban Environmental Processes and Pollution Control, CAS Haixi Industrial Technology Innovation Center in Beilun, Ningbo 315830, China; d School of Life Sciences, Hebei University, Baoding 071000, Hebei, China; e College of Science and Engineering, Flinders University, Bedford Park, South Australia, Australia; f The Aerobiome Innovation and Research Hub, College of Science and Engineering, Flinders University, Bedford Park, South Australia, Australia

**Keywords:** One health, Antibiotic intervention, Free-roaming cats, Zoonotic bacterial pathogens

## Abstract

Free-roaming cats are abundant in many urban ecosystems but pose significant public health and ecological risks as potential reservoirs of potential zoonotic pathogens. This study examined the gut microbiome of free-roaming cats in urban green spaces in Beijing, China, focusing on microbial diversity and potential zoonotic pathogen prevalence. Using 16S rRNA amplicon sequencing and the MBPD zoonotic pathogen detection pipeline, we identified multiple opportunistic zoonotic bacterial pathogens, including *Clostridium perfringens*, *Pseudomonas stutzeri*, *Enterobacter cloacae*, *Aeromonas veronii*, *Klebsiella pneumoniae*, *Escherichia coli*, *Stenotrophomonas maltophilia*, and *Streptococcus salivarius* (including ESKAPE pathogens). Antibiotic treatment was associated with reduced microbial diversity (Shannon index: 4.1–5.5 in non-treated cats vs. 1.9–4.7 in treated cats) and a lower relative abundance of key pathogens, including *E. cloacae* and *K. pneumoniae*. However, we observed a paradoxical shift in microbiome structure: in non-treated cats, higher microbial diversity was associated with lower pathogen abundance, whereas in treated cats, diversity positively correlated with pathogen presence, possibly due to microbiome instability or opportunistic recolonisation after antibiotic disruption. These findings highlight the complex trade-offs of antibiotic-driven microbiome disruption and highlight the need for microbiome-informed One Health interventions. Given the role of microbial diversity in pathogen suppression, alternative strategies—such as probiotic supplementation—may offer a more sustainable approach to mitigating zoonotic disease transmission while balancing urban biodiversity conservation and public health safety.

## Highlights

• Free-roaming urban cats harbour diverse gut microbiota, including multiple zoonotic pathogens (incl. *Klebsiella pneumoniae*).
• Antibiotics can reduce microbial diversity and pathogen abundance, but destabilise community structure in a deleterious manner.
• Alternative One Health approaches may be needed, such as probiotics, to mitigate zoonotic risks sustainably.

## 1 Introduction

Cats have shared a long and complex history with human societies, serving various roles from pest controllers to companions [1]. These felines, particularly free-roaming cats, have become common in urban ecosystems. However, the proliferation of free-roaming cats in urban areas is a growing concern due to abandonment, stray populations, supplementary feeding practices and their prolific reproductive abilities [[2], [3]]. This increase in free-roaming cat populations presents significant public health and ecological challenges, particularly in the context of zoonotic disease transmission [[4], [5]].

Zoonotic diseases, which can be transmitted between non-human animals and humans, represent a critical One Health challenge [6]. Notably, at least 70 % of emerging zoonoses originate from wildlife, often emerging at human-wildlife interfaces [7]. Free-roaming cats are key players in this dynamic, as potential reservoirs for various pathogens. Previous studies have identified cats as hosts for zoonotic agents such as *Brucella* sp., *Bartonella henselae*, and *Leptospira* sp. [[8], [9]]. Moreover, the risk of *Toxoplasma gondii* transmission by free-roaming cats in university settings has been documented [10]. The Trap-Neuter-Return (TNR) intervention aims to humanely reduce the number of urban free-roaming cats by advocating for neutering instead of euthanasia [11]. However, antibiotic treatments associated with TNR may have unintended consequences for microbiome stability and pathogen dynamics in treated cats. Because antibiotic administration exerts profound effects on host-associated microbial communities by reshaping their structure and ecological dynamics [12]. Beyond targeting pathogenic bacteria, antibiotics often disrupt commensal taxa, leading to marked alterations in microbiome composition and reductions in overall diversity [13]. Such disturbances can diminish colonization resistance, thereby creating ecological niches that favor the expansion of opportunistic or antibiotic-resistant species [14]. Consequently, antibiotic treatment not only influences the trajectory of pathogen suppression or persistence but also modulates long-term community recovery and host–microbe interactions.

Urban green spaces provide sheltered environments that often support stable populations of free-roaming cats [15]. University campuses, in particular, offer abundant resources, including prey (such as birds and rodents) and anthropogenic food sources [[16], [17]]. The frequent interactions between cats and students, driven by the animals' appealing nature, further elevate public health concerns in these settings [18]. In China, universities, functioning as semi-enclosed areas with clearly defined administrative boundaries, often support relatively stable free-roaming cat populations. These environments provide valuable microcosms for studying urban ecological interactions and associated health risks [18]. While previous research has shed light on specific pathogens associated with free-roaming cats, a comprehensive analysis of the bacterial pathogens carried by these animals in urban environments remains lacking. This knowledge gap is particularly concerning given the risk of zoonotic disease transmission in densely populated areas and the broader implications for urban environmental health.

Our study aims to address this gap by characterising the zoonotic bacterial pathogens harboured by free-roaming cats in urban green spaces in Beijing, China. We hypothesize that:

1) The gut microbiome of free-roaming cats harbours a diverse array of bacteria and bacterial potential zoonotic pathogens.
2) Antibiotic intervention reduces the relative abundance of potential zoonotic pathogens in cat guts and therefore the zoonotic risk, and associates with a shift in overall gut microbial composition.

By elucidating the microbial ecology of free-roaming cats and the effects of antibiotic interventions, this research contributes to evidence-based zoonotic disease mitigation strategies for urban wildlife management.

## 2 Materials and methods

### 2.1 Sample collecting and processing

We conducted a comparative study of the gut microbiome in free-roaming cats across ten university campuses in Beijing City. These universities were selected based on their distinct boundaries and relatively stable population composition, providing controlled environments for our study (detailed site characteristics are provided in Supplementary (Supplementary Tbale 1)). A total of 29 fresh fecal samples were collected from two groups (Supplementary Table 2):

1) Non-treated group: 16 samples from free-roaming cats with no medical intervention for at least six weeks.
2) Treated group: 13 samples from cats that had undergone antibiotic intervention during the Trap-Neuter-Return (TNR) using Shotapen L.A., a long-acting penicillin-class antibiotic (the Treated Group).

The non-treated group comprised cats living freely on university campuses without medical intervention for half a year. For the treated group, fecal samples were collected from cats that had undergone antibiotic intervention during Trap-Neuter-Return (TNR), which is part of the veterinary protocol. In TNR, Shotapen L.A. is commonly used, often considered standard practice, precisely because the cats cannot be monitored or treated after release. Preventing an infection is far easier and safer than trying to treat one in a free-roaming cat. A single long-acting shot protects for several days (typically 3–5 days) during the critical initial healing phase. Only in some rare cases, Shotapen L.A. can cause severe allergic reactions. A single injection is given under the skin (subcutaneously) by the veterinarian during the surgical procedure, usually right after the cat is anesthetized. Dosage was based on weight. A common dose is 0.1 mL per kg (or 1 mL per 10 kg / 22 lbs). The cats included in our study received no intervention stemming from the experiment itself. The institutional animal ethics committee confirmed that formal approval was not required for observational and non-invasive fecal sample collection of free-roaming cats in our study.

We collected only fresh, moist fecal specimens after excretion within 24 h. The stools were sampled after antibiotic intervention at least six hours based on the cat's life habits. Each sample was individually collected using 50 mL Falcon tubes to prevent cross-contamination. Samples were immediately placed in sterile containers and transported to the laboratory under dry ice and foam box conditions within 24 h of collection. Upon arrival at the laboratory, all samples were stored at −20 °C until further processing.

### 2.2 DNA extraction and 16S gene amplicon sequencing

The Fast DNA Spin Kit for soil (MP Biomedicals, USA) was used to extract DNA from approximately 0.25 g of feces, according to the manufacturer's instructions. A Qubit 3.0 fluorimeter (Invitrogen) was used to quantify the extracted DNA quality and concentration before storing it at −20 °C until further analysis.

The fecal DNA was used to analyze bacterial diversity and composition. To characterize the diversity and community composition of bacteria, Polymerase chain reaction (PCR) amplification was done in triplicate using the 338F/806R primer targeting the V3–V4 region of the 16S rRNA gene (5′-CCTAYGGGRBGCASCAG-3′/5′-GGACTACNNGGGTATCTAAT-3).

The 300-bp paired-end run was sequenced via Illumina Miseq PE300 (Majorbio, Shanghai, China). Species abundance at >0.01 % reads was considered present in a sample. The obtained sequences were checked using DADA2, and low-quality sequences were discarded. The resulting high-quality sequences were analyzed using Quantitative Insights Into Microbial Ecology version 2 (QIIME2). ASVs were generated, and ASVs with only one sequence were discarded in the following analysis. Silva (v138) was used to classify the bacterial taxa with a Naïve Bayes classifier. ASVs identified as mitochondria and chloroplast sequences were removed. The checked ASVs were used to calculate α-diversity (Shannon and Chao1 indices) of microbial communities using QIIME2.

### 2.3 Data analysis and statistical analysis

Data analyses were performed in R Studio (v.4.0.5). Most of the results were computed and visualized by the R package “microeco” (Principal Coordinates Analysis, stacked bar chart of bacteria relative abundance, and LefSe analysis) [19]. A recent multi-bacteria database pipeline (MBPD) was used to identify potential zoonotic bacterial pathogens in the composition of the gut microbiome of free-roaming cats [20]. We calculated the relative abundance of zoonotic pathogens by the ratio of ASV numbers to the total number of sequences from “seqNum.txt” in the pipeline output files.

The online analysis platform Wekemo Bioincloud (https://www.bioincloud.tech) was used to visualize the microbial co-occurrence network, use the Spearman correlation coefficient, and correct for false discovery rate [21]. The co-occurrence networks were generated to compare the differences in bacterial community complexity between the treated and non-treated groups. The topological features of the networks were calculated to estimate the complexity of the bacterial co-occurrence network between treated and non-treated groups, including average degree, density, average closeness centrality and average betweenness centrality. Here, average degree refers to the average number of edges (links) of each node, clustering coefficient refers to the degree to which the nodes tend to cluster together, average closeness centrality and average betweenness centrality represent the intensity of connections among nodes, and modularity measures how separated the modules of nodes are from each other [22]. Therefore, higher average degree, average closeness centrality, and low average betweenness centrality suggest a higher complexity of the microbial network [23].

Linear discriminant analysis effect size (LefSe) analyses were used to identify different bacterial features among the non-treated group (*n* = 16) and the treated group (*n* = 13) (LDA score > 3.5). We used the R package “vegan” for statistical analysis [24]. The Kruskal−Wallis H test with post-hoc test was used for multiple comparisons, for two-group comparisons, a two-tailed Wilcoxon (paired) or Mann−Whitney *U* test (unpaired) test was used.

## 3 Results

### 3.1 Prevalence and distribution of potential zoonotic pathogens and the impacts of antibiotics

**Fig. 1:**
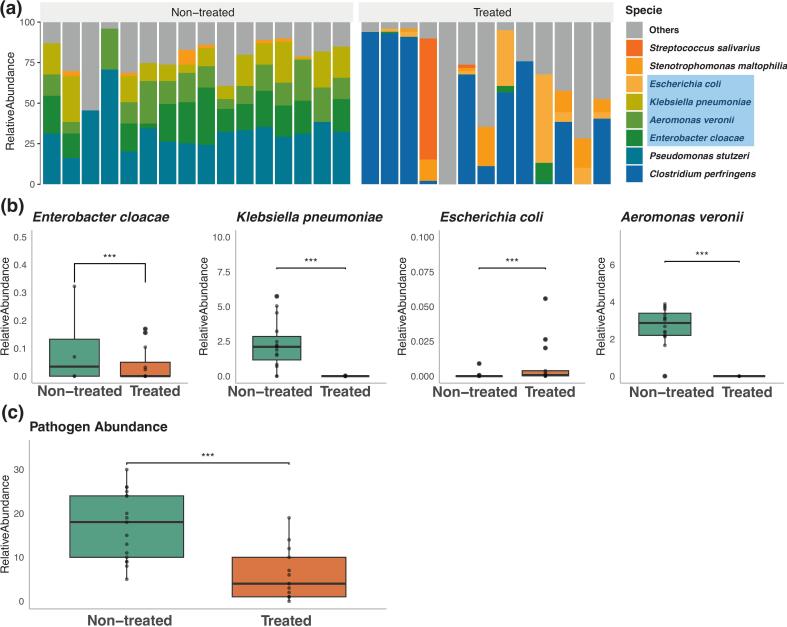
Potential zoonotic pathogens in the gut microbiome of free-roaming cats. (a) Relative abundance of the 8 most prevalent zoonotic bacterial pathogen species, with emphasis on Aeromonadaceae and Enterobacteriaceae. (b) Comparison of relative abundances of four critical bacterial pathogens (*E. cloacae*, *K. pneumoniae*, *E. coli*, and *A. veronii*) between non-treated and treated groups. (c) Overall relative abundance of potential zoonotic bacterial pathogens in non-treated versus treated groups (Kruskal-Wallis post-hoc test, *P* < 0.05).

A total of 29 fresh fecal samples were collected from treated and non-treated groups. The treated group consisted entirely of individuals under 12 months of age. In the non-treated group, 75 % of individuals were under 12 months old. The treated group included 9 females and 4 males, while the non-treated group comprised 8 females and 8 males. Analysis of the gut microbiome revealed several opportunistic potential zoonotic bacterial pathogens across all samples (Fig. 1a). The eight most prevalent species identified were *Clostridium perfringens*, *Pseudomonas stutzeri*, *Enterobacter cloacae*, *Aeromonas veronii*, *Klebsiella pneumoniae*, *Escherichia coli*, *Stenotrophomonas maltophilia*, and *Streptococcus salivarius*. Four species of particular concern - *E. cloacae*, *K. pneumoniae*, *E. coli*, and *A. veronii* - showed markedly higher relative abundance in the non-treated group compared to the treated group (Fig. 1b). Specifically, *E. cloacae* (0.04 % vs 0 %), *K. pneumoniae* (2.13 % vs 0 %), *E. coli* (0.007 % vs 0 %), and *A. veronii* (2.86 % vs 0 %) were significantly more abundant in the non-treated group (Kruskal-Wallis post-hoc test, *P* < 0.05).

The overall relative abundance of potential zoonotic bacterial pathogens was significantly higher in the non-treated group compared to the treated group (Fig. 1c, Kruskal-Wallis post-hoc test, *P* < 0.05).

### 3.2 Gut microbiome composition and diversity of free-roaming cats

**Fig. 2:**
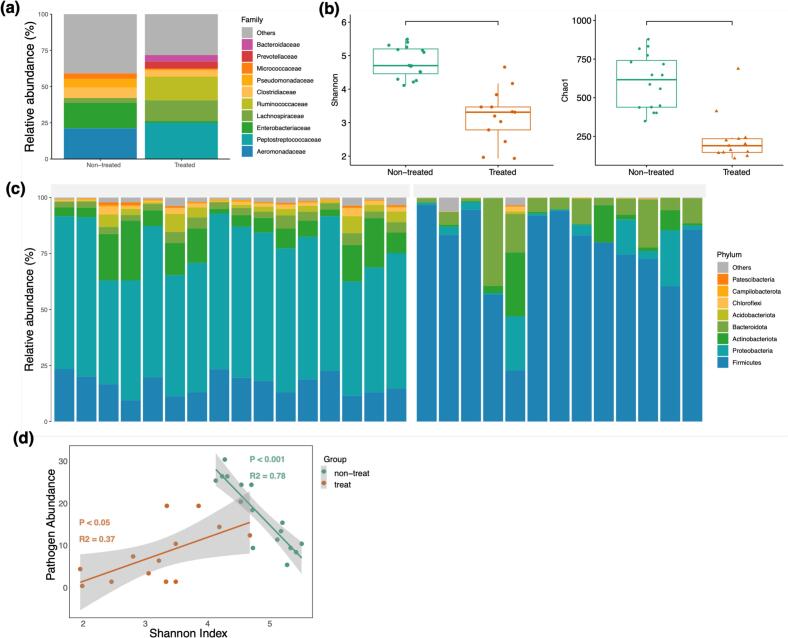
Microbiome profiles of non-treated and antibiotic-treated free-roaming cat populations. (a) Relative abundance of the 8 most dominant bacterial families in each group. (b) Alpha-diversity comparison using Shannon and Chao1 indices (Kruskal-Wallis post-hoc test, *P* < 0.05). (c) Relative abundance of the 8 most dominant bacterial phyla across all samples. (d) Linear regression model depicting the relationship between the relative abundance of zoonotic pathogens and Shannon diversity index of the gut microbiome.

The four dominant bacterial phyla in the non-treated group were Firmicutes, Proteobacteria, Actinobacteriota and Bacteroidota. At the family level, Aeromonadaceae and Enterobacteriaceae, both belonging to the Proteobacteria phylum, were the predominant bacteria (Fig. 2a and Fig. 2b). In the treated group, the composition of the four dominant bacterial phyla remained the same as in the non-treated group; however, the relative abundance of Firmicutes was significantly higher, while that of Proteobacteria was lower than non-treated group. (Fig. 2a and Fig. 2b). At the family level, Peptostreptococcaceae, Lachnospiraceae, and Ruminococcaceae emerged as the three dominant bacterial families.

Alpha diversity metrics showed distinct patterns between the two groups (Fig. 2c). The Shannon index ranged from 4.1 to 5.5 in the non-treated group and 1.9 to 4.7 in the treated group. Similarly, the Chao1 index spanned from 348.9 to 878.4 in the non-treated group and 107.83 to 687.6 in the treated group. Both indices were significantly lower in the treated group (Kruskal-Wallis post-hoc test, *P* < 0.05), indicating a lower bacterial richness and evenness in treated cats. Strikingly, linear regression analysis revealed a negative correlation between the Shannon diversity index and the relative abundance of zoonotic pathogens in the non-treated group (R^2^ = 0.78, *P* < 0.001), while a positive correlation was observed in the treated group (R^2^ = 0.37, *P* < 0.05) (Fig. 2d).

### 3.3 The different gut microbial composition between antibiotic-treated and non-treated groups

**Fig. 3:**
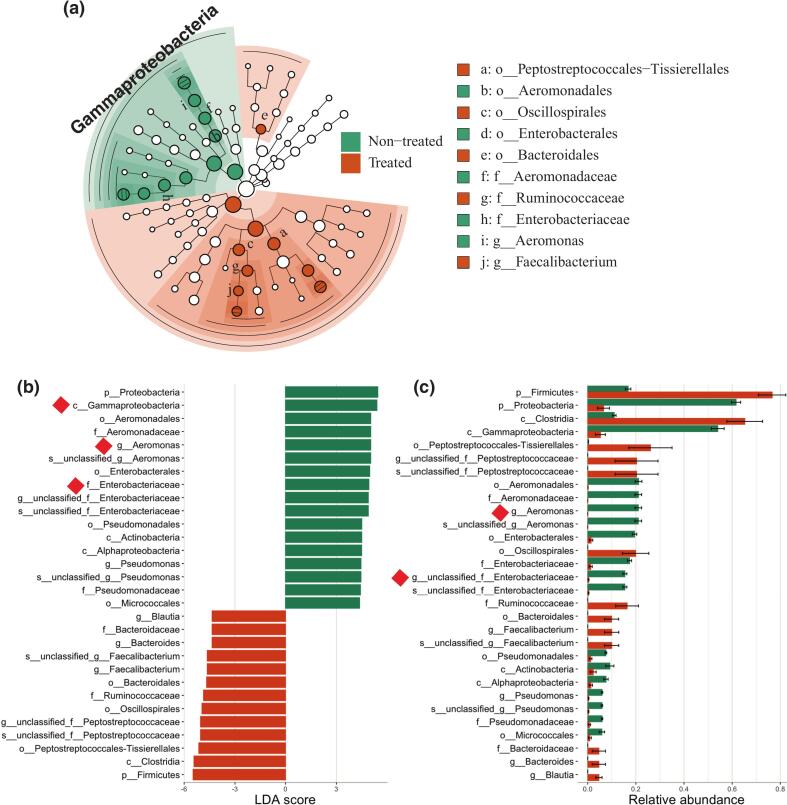
Differential abundance analysis of microbial taxa between non-treated and antibiotic-treated groups. Cladogram based on Linear Discriminant Analysis Effect Size (LEfSe) showing significant differences in taxa from class to genus level (LDA score > 3.5).

LEfSe analysis revealed significant biomarkers in the non-treated and treated groups (Fig. 3). In the non-treated group, Gammaproteobacteria was significantly abundant at the class level (LDA score > 3.5, *P* < 0.05), indicating its strong association with the non-treated condition (Fig. 3a). Several genera belong to Gammaproteobacteria exhibited higher relative abundance in the non-treated group than the treated group (Fig. 3b). For example, Aeromonadaceae and Enterobacteriaceae were significant biomarkers at the family level in the non-treated group (LDA score > 3.5, *P* < 0.05). Notably, Aeromonas and unclassified_Enterobacteriaceae genera showed significantly higher abundance in the non-treated group (Kruskal-Wallis post-hoc test, *P* < 0.05). The LDA scores for these genera were among the highest, emphasizing their potential importance as discriminative features in the non-treated group.

In contrast, the treated group was characterized by a higher abundance of certain Firmicutes taxa (Fig. 3c). Specifically, genera belonging to the Clostridia class, such as Romboutsia and unclassified_Peptostreptococcaceae, were significantly enriched in the treated group (LDA score > 3.0, *P* < 0.05). This shift towards Firmicutes dominance in the treated group aligns with the phylum-level observations (Fig. 2c). These findings highlight the substantial impact of antibiotic treatment on the microbial community structure, particularly the reduction of potentially pathogenic Gammaproteobacteria and the concurrent increase in certain Firmicutes taxa. The consistent pattern observed across multiple taxonomic levels (from class to genus) reinforces the robustness of these microbial shifts associated with antibiotic intervention in free-roaming cats.

### 3.4 Antibiotic-induced alterations in microbial co-occurrence networks

**Fig. 4:**
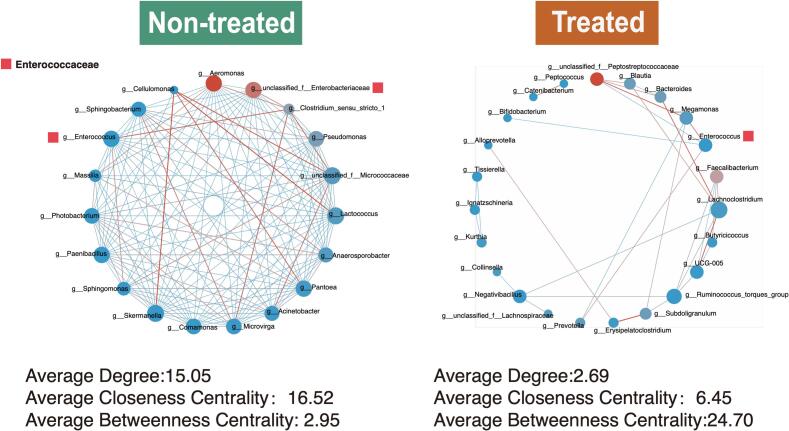
Microbial co-occurrence networks in non-treated and antibiotic-treated groups. Network visualization based on Spearman correlation coefficients (*P* < 0.05). Node colors represent the summed relative abundance of taxa. Key differences in network topology and interactions between the two groups are highlighted.

In the microbial co-occurrence networks of the non-treated group, potential pathogen-associated bacteria, *Aeromonas*, *Enterococcus*, and *unclassified_Enterobacteriaceae* occurred in important nodes with high relative abundance (Fig. 4). The *unclassified_Enterobacteriaceae* and *Enterococcus* showed positive correlations with *Clostridium* sensu stricto *1*, suggesting a potentially important ecological interaction.

The treated group network was characterized by positive correlations between *Enterococcus*, *Megamonas*, and *Prevotella*, and negative correlations with *Alloprevotella* and *unclassified_Peptostreptococcaceae*. The *Aeromonas* and *unclassified_Enterobacteriaceae* disappeared in the network (Fig. 4). The positive connection between *Enterococcus*- *Clostridium* sensu stricto *1 was* also dismissed, indicating a restructuring of the microbial community following antibiotic intervention.

Network metrics differed substantially between groups. The average degree was 15.05 in the non-treated group and 2.69 in the treated group, indicating a reduction in overall network connectivity post-treatment. Average closeness centrality values were 16.52 and 6.45, while average betweenness centrality values were 2.95 and 24.70 for non-treated and treated groups, respectively. These changes in network properties suggest a significant reorganization of microbial interactions following antibiotic treatment.

## 4 Discussion

Our study reveals that urban free-roaming cats may pose a health risk to people due to their role as reservoirs of bacterial pathogens, including the ESKAPE pathogen *Klebsiella pneumoniae* [[25], [26]]. This finding is particularly relevant in urban green space environments, where close human-animal interactions are common. Similar concerns have been raised about the role of urban free-roaming cats as vectors for other zoonotic diseases in densely populated areas [27].

Significant alterations in the gut microbiome of free-roaming cats following antibiotic intervention have important implications for urban environmental health and public safety. The impact of antibiotic treatment on microbial diversity, composition, and community structure in these felines highlights the complex interplay between management interventions and ecological consequences in urban environments. The observed reduction in potential zoonotic pathogens, particularly *E. cloacae*, *K. pneumoniae*, *E. coli*, and *A. veronii*, in treated cats suggests that antibiotic intervention may decrease the risk of zoonotic disease transmission. However, the benefits of pathogen reduction must be weighed against the potential for antibiotic resistance development, a growing concern in both human and veterinary medicine [28].

The contrasting relationships between microbial diversity and pathogen abundance in treated and non-treated groups provide valuable insights into the ecological dynamics of the feline gut microbiome. Strikingly, higher microbial diversity in non-treated cats was associated with lower pathogen abundance, suggesting that a diverse microbiome may offer protection against colonization by potential pathogens. This aligns with the findings of Buffie et al. [29], who reported similar protective effects of microbial diversity in human gut microbiomes. This suggests that an effective strategy could be to trap and neuter free-roaming cats and simultaneously enhance their gut microbial diversity (e.g., giving them pre- and probiotics rather than antibiotics). This could be a favourable alternative given the negative impacts of antimicrobial-resistant genes in the environment and the potential for long-term adverse health impacts on the cats [[30], [31]]. We can speculate that these adverse health impacts could reduce the condition of the cats and, in turn, make them shed more pathogens.

The reversal of this relationship in treated cats indicates that reducing specific bacterial taxa disrupts these protective ecological interactions, potentially creating opportunities for pathogen colonization during microbiome recovery. In treated cats, the positive correlation could be due to the recovery dynamics where some species rapidly rebound and fill niches, including both harmless commensals and resistant pathogens, leading to increased diversity and pathogen presence.

Previous studies revealed the fundamental ecological interactions of specific taxa in microbial communities [[32], [33]]. The results of substantial changes observed in microbial co-occurrence networks following antibiotic treatment demonstrate that antibiotic intervention alters the abundance of specific taxa and fundamentally reduces the interaction degree between the potential pathogens and non-pathogenic gut bacteria in the gut microbiome of free-roaming cats. The reconstruction of the treated cat network also indicates a fundamental restructuring of microbial communities. The reduced network connectivity in treated cats suggests a simplification of microbial interactions, which could impact the resilience and stability of the gut ecosystem. Similar network alterations have been reported in human studies following antibiotic treatment [34]. The loss of specific microbial associations, such as the positive correlations between Enterobacteriaceae and *Clostridium* sensu stricto *1* in non-treated cats, may have functional consequences for the host, potentially affecting metabolic processes and immune regulation [35]. These findings have significant implications for urban wildlife management. While antibiotic intervention may reduce immediate health risks associated with zoonotic pathogens, it also alters the complex microbial ecosystems that may play protective roles. This highlights the complex trade-offs in the dynamic regulation of environmental health during antibiotic intervention practices.

The antibiotic treatment, often implemented by Shotapen L.A., a penicillin-class antibiotic, should consider the broader ecological impacts of these interventions. Future urban wildlife management strategies should aim to balance reducing zoonotic disease risks with maintaining beneficial microbial communities. This may involve developing targeted interventions that specifically address high-risk pathogens while minimizing disruption to the overall microbiome. Additionally, long-term monitoring of treated populations could provide valuable insights into the resilience and recovery of feline gut microbiomes following intervention. As alluded to, it might also be worthwhile investigating the role of pre- and probiotics in enhancing the free-roaming cat gut microbiome to reduce the prevalence of specialised and opportunistic zoonotic pathogens, rather than using antibiotics.

The university setting as part of urban green space offers a unique opportunity to study these dynamics, given the semi-closed nature of campus environments and the stable cat populations they often support [18]. However, it also presents specific challenges due to frequent human-non-human animal interactions. Education programs for students and staff about safe interactions with free-roaming cats, coupled with evidence-based management strategies informed by microbiome research, could help mitigate potential health risks while fostering a balanced urban ecosystem.

Our study has two limitations. First, we collected feces from treated and non-treated cats rather than longitudinal samples (before and after treatment) from the same cats. Therefore, our conclusions are limited to suggesting potential ‘antibiotic’ influences, rather than establishing definitive causality. Second, our investigation involved only one antibiotic (Shotapen L.A.) commonly used in feline clinical practice. Since gut microbial communities exhibit varied responses to different antibiotics, future studies should investigate the effects of various antibiotic interventions on the feline gut microbiome and zoonotic pathogenic bacteria. Third, both the stress induced by the TNR procedure and the effects of antibiotic intervention may alter the cat's microbial composition and diversity, and their combined impact warrants further investigation.

## 5 Conclusion

Our study underscores the critical intersection between urban wildlife management, microbial ecology, and public health through a One Health lens. By examining how antibiotic interventions shape the gut microbiomes of free-roaming cats, we provide evidence for the unintended ecological consequences of pathogen control measures in urban environments. Given the interconnected health risks posed by zoonotic pathogens, future strategies should move beyond antibiotic-based interventions and explore microbiome-informed approaches that balance animal, environmental, and human health. The potential for probiotic or prebiotic supplementation as a sustainable alternative to antibiotics warrants further investigation, as it may help stabilize microbiomes while reducing zoonotic transmission risks. A One Health approach that integrates microbial surveillance, targeted interventions, and long-term ecosystem monitoring is essential for managing urban free-roaming animals in a way that safeguards biodiversity, promotes animal welfare, and mitigates public health threats.

## Authors contribution

X.S: conceptualization, project administration, resources, supervision, writing–review & editing. A.X, Y.Y.Z: conceptualization, data curation, visualization, writing–original draft, writing–review & editing. Z.H.T: visualization. J.M.R, Q.S·H, J.Q.S: writing–review & editing.

## Declaration of competing interest

The authors declare that they have no known competing financial interests or personal relationships that could have appeared to influence the work reported in this paper.

## Data Availability

Data will be made available on request.

## Appendix A Supplementary data

Supplementary material 1: The sampling sites of this study Image 1

Supplementary material 2: The metadata of free-roaming cats in this study Image 2
